# The Incubation Period of Buruli Ulcer (*Mycobacterium ulcerans* Infection)

**DOI:** 10.1371/journal.pntd.0002463

**Published:** 2013-10-03

**Authors:** Jason A. Trubiano, Caroline J. Lavender, Janet A. M. Fyfe, Simone Bittmann, Paul D. R. Johnson

**Affiliations:** 1 Department of Infectious Diseases, Austin Health, Heidelberg, Victoria, Australia; 2 Victorian Department of Health, Melbourne, Victoria, Australia; 3 Victorian Infectious Disease References Laboratory (VIDRL), North Melbourne, Victoria, Australia; 4 World Health Organization Collaborating Centre for Mycobacterium ulcerans (Western Pacific Region), VIDRL, North Melbourne, Victoria, Australia; 5 Department of Medicine, Austin Health, University of Melbourne, Heidelberg, Victoria, Australia; 6 Department of Microbiology & Immunology, University of Melbourne, Parkville, Victoria, Australia; University of California San Diego School of Medicine, United States of America

## Abstract

**Introduction:**

Buruli Ulcer (BU) is caused by the environmental microbe *Mycobacterium ulcerans*. Despite unclear transmission, contact with a BU endemic region is the key known risk factor. In Victoria, Australia, where endemic areas have been carefully mapped, we aimed to estimate the Incubation Period (IP) of BU by interviewing patients who reported defined periods of contact with an endemic area prior to BU diagnosis.

**Method:**

A retrospective review was undertaken of 408 notifications of BU in Victoria from 2002 to 2012. Telephone interviews using a structured questionnaire and review of notification records were performed. Patients with a single visit exposure to a defined endemic area were included and the period from exposure to disease onset determined (IP).

**Results:**

We identified 111 of 408 notified patients (27%) who had a residential address outside a known endemic area, of whom 23 (6%) reported a single visit exposure within the previous 24 months. The median age of included patients was 30 years (range: 6 to 73) and 65% were male. 61% had visited the Bellarine Peninsula, currently the most active endemic area. The median time from symptom onset to diagnosis was 71 days (range: 34–204 days). The midpoint of the reported IP range was utilized to calculate a point estimate of the IP for each case. Subsequently, the mean IP for the cohort was calculated at 135 days (IQR: 109–160; CI 95%: 113.9–156), corresponding to 4.5 months or 19.2 weeks. The shortest IP recorded was 32 days and longest 264 days (Figure 1 & 2). IP did not vary for variables investigated.

**Conclusions:**

The estimated mean IP of BU in Victoria is 135 days (IQR: 109–160 days), 4.5 months. The shortest recorded was IP 34 days and longest 264 days. A greater understanding of BU IP will aid clinical risk assessment and future research.

## Author Summary

Buruli Ulcer (BU) is a necrotizing skin infection caused by the environmental organism *Mycobacterium ulcerans*. Despite an unknown mode of transmission, contact with a BU endemic region is the known key risk factor. In Victoria, Australia, endemic areas have been carefully mapped and patients are known to report single visits or defined exposures to these areas. From a retrospective review, we identified 23 patients with a single visit exposure to BU endemic regions from a total of 408 notifications of BU in Victoria (2002–2012). We were able to estimate the time from exposure to disease onset, incubation period (IP), via notification record review and telephone questionnaire. The IP of the cohort was subsequently estimated at 4.5 months (Range 1–9 months). An understanding of the IP of BU will be of value to communities, clinicians and government health bodies via aiding clinical risk assessment, epidemiological studies and public health investigations in the future.

## Introduction

*Mycobacterium ulcerans*, a slow growing environmental pathogen [1], causes a necrotizing cutaneous infection with various names but now known internationally as Buruli Ulcer (BU). The mode of transmission remains controversial [2] [3] however the key identifiable risk factor for BU is residence in or contact with a Buruli endemic region. Worldwide, most cases of BU occur in patients who live permanently in endemic areas, and because the mode of transmission is unknown it is generally impossible to estimate the incubation period (IP).

The first definitive description of *M. ulcerans* infection (termed Bairnsdale ulcer in that report) was in Australia in 1948 [1] [4]. Cases have been reported from at least 32 countries, in regions including Africa, Australia, Southeast Asia, China, Central and South America and the Western Pacific [5]. In Victoria, cases have been reported in Gippsland, Phillip Island [6] and the Mornington [7] and Bellarine Peninsulas [8] [9] [10]. In Queensland there is a significant established focus in Far North Queensland [11] [12]. Sporadic cases have been reported in the Northern Territory (NT) [13] and Capricorn Coast of southern Queensland [14]. Single cases have occurred in Western Australia (WA) [15] and New-South Wales (NSW) [16]. Transmission is not reported in South Australia or Tasmania.

Few published studies exist dedicated to the incubation of BU. The Uganda Buruli Group estimated that the normal IP was 4–13 weeks [17]. The shortest IP described is 2–3 weeks, in a newborn from Papua New Guinea [18]. Recently within Australia, five patients living outside endemic areas with travel to known endemic regions had reported incubation periods ranging from 2 to 5 months [19]. In Victoria where BU is legally notifiable, diagnosis by Polymerase Chain Reaction (PCR) is centralized and rapid and endemic areas are systematically mapped by members of the WHO Collaborating Centre (WHOCC) in Melbourne. Hence there is a unique opportunity to systematically estimate IP.

## Methods

BU has been a legally notifiable infection by treating physicians and laboratories in Victoria since January 2004 and data has been collected systematically and recorded for several years before this. For this investigation we reviewed notifications in the 10-year period 2002 and 2012. The case definition for BU was the presence of a lesion clinically suggestive of *M. ulcerans* infection together with at least one of;

- Positive polymerase chain reaction (PCR) for IS*2404* [20] [21] [22]
- Culture and specific identification of *M. ulcerans* by a Mycobacterium Reference Laboratory.

**Figure 1:**
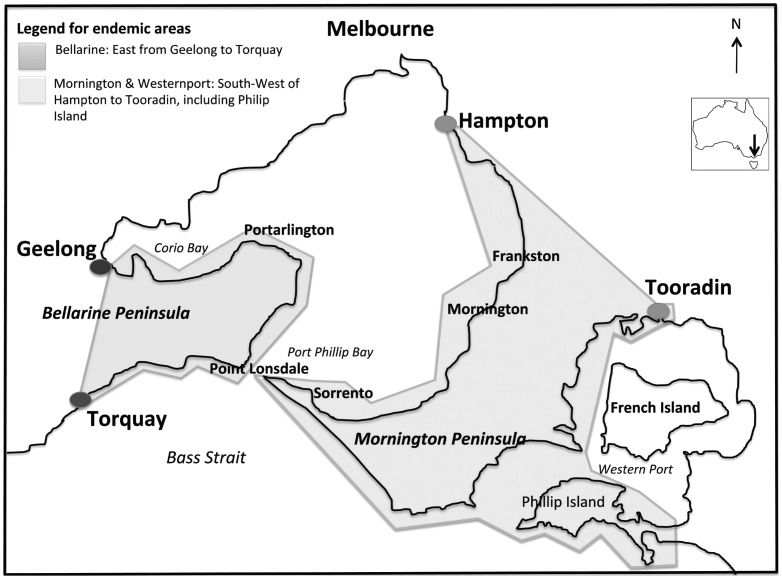
Geographic representation of Bellarine Peninsula, considered endemic for BU as of 2012. Bellarine Peninsula – east of line from Geelong to Torquay. Mornington and Westernport – southwest of line from Hampton to Tooradin (including Phillip Island).

**Figure 2:**
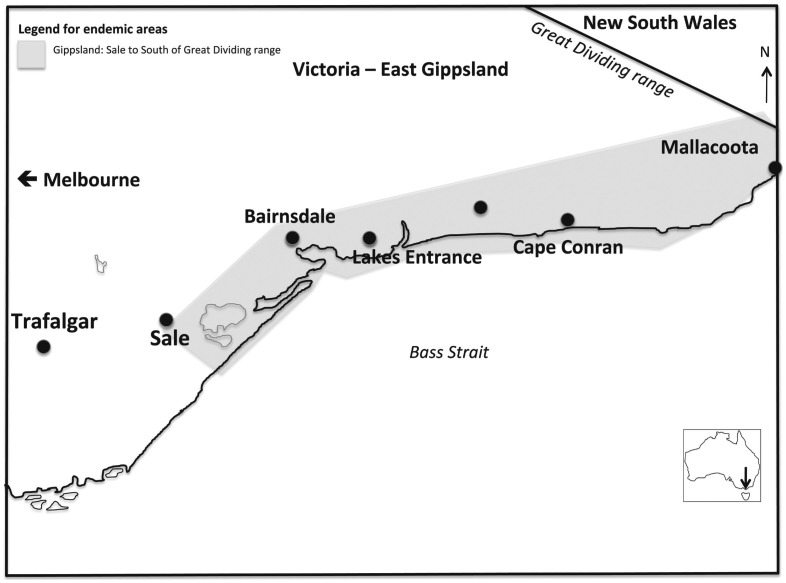
Geographic representation of East Gippsland, considered endemic for BU as of 2012. East Gippsland: East of Sale and south of the great divide.

An endemic region was defined as the constant presence of the organism (e.g. *M. ulcerans*) within a given geographic area or population group [23]. Following literature review and consultation with the Health Department records and those of the WHOCC, endemic regions within Australia were defined and mapped (Table 1, Figure 1 & 2). Significant exposure was defined as greater than one continuous hour in an endemic area and the maximum possible IP considered was two years prior to diagnosis.

**Table 1:** Regions within Eastern Australia defined as endemic for *M.ulcerans*.

State	Regions
*Victoria (VIC)* ^[27]^	Melbourne Bayside ^[7][9]^ East Gippsland ^[4][1]^ Westernport ^[7][6][28]^ Bellarine Peninsula ^[9][10][29][8][2]^ Mornington Peninsula ^[7][28]^
*North Queensland (NQLD)* ^[14][11][12]^	Daintree locality, Daintree River & adjacent coastal lowlands, Mossman Central coast
*New South Wales (NSW)* ^[16]^	Southern coastal regions

Telephone interview with a structured questionnaire was undertaken with cases or next-of-kin in conjunction with review of medical records and Victorian Department of Health notification record. Data were collected for all geographical exposures to designated BU endemic areas and a map was provided to interviewees to assist them. The duration of exposure was obtained for a single visit and date of onset of symptoms recorded. If a patient was unable to recall a single date of disease exposure, a period of exposure was obtained (maximum one month) and a minimum and maximum IP value for that patient calculated (IP range). If a patient had multiple exposures to known endemic regions within Australia (Northern Territory, Queensland, Western Australia) or overseas and only one occurring within Southern Australia (Victoria and NSW), where possible, variable-number tandem repeat (VNTR) typing was performed on the isolate to identify the region of origin [19] [24]

For the 23 patients with a single visit exposure, an IP range was calculated in days and months. The midpoint of the IP range was utilized to calculate a point estimate of the IP for each case. Subsequently, a mean IP for the entire study cohort was calculated. Baseline patient characteristics and data collected were patient residential address, age, sex, location of lesion, duration of endemic exposure, date of first medical attention/form of medical attention, date of when BU first suspected, specimen provided for diagnosis, PCR confirmation, treatment and outcome(s).

### Ethics statement

The *M. ulcerans* investigation was performed as part of an ongoing enhanced surveillance project through the Victorian Department of Health, Victoria, Australia under its responsibilities to record and investigate notifiable diseases. As such interviews of notified cases of BU are routinely conducted, additional ethics approval was not required. No informed patient consent was required.

### Statistical analyses

Statistical analyses were performed using STATA version 10.0 (STATA Corporation, College Station, Rx) and PRISM graphpad software 2012.

## Results

Of the 408 notifications of BU in the 10-year period 2002–2012, 111 (27%) patients had residential addresses outside the assigned endemic regions, 23 (6%) with single visit exposures and it was from these patients that the IP was estimated. The median age at time of diagnosis of this group was 30 years (Range: 6–73), 65% were male. The residential addresses of all patients were outside established endemic regions (Figure 1 & 2). The endemic areas visited were the Bellarine Peninsula (14; 61%), Phillip Island (3; 13%) and Mornington Peninsula (3; 13%), with single visit exposures to each of Gippsland (VIC), Darwin (NT) and Port Douglas/Mossman (QLD). The duration of exposure was predominately greater than 7 days (60%), but single day exposure was noted in 4 patients (17%) (Table 2).

**Table 2:** Characteristics of the 23 cases of BU included in the study cohort.

Characteristic (N = 23)	No. (%)	
*Median Age* (6–73 years)	30 years	
0–10 years	4	17
10–18 years	6	26
18–30 years	2	9
30–60 years	6	26
>60 years	5	22
*Notification date*		
2003–2006	6	25
2007–2009	4	17
2010–2012	13	58
*Sex*		
Male	15	65
Female	8	35
*Endemic Area Exposed* a		
Bellarine	14	61
Mornington	3	13
Phillip Island	3	13
Gippsland	1	4
NT	1	4
NQLD	1	4
*Season of exposure*		
Summer	14	61
Autumn	4	17
Winter	3	13
Spring	2	9
*Duration of exposure (days)*		
Single	4	17
1–7	6	26
7–14	2	9
>14	11	48
*Mosquito bites during exposure*		
Yes	13	57
No	8	35
Unknown	2	9
*Open wounds during exposure*		
Yes	1	4
No	22	96
*Location of lesion*		
Thigh	1	4
Knee	3	13
Calf	7	30
Ankle	7	30
Shoulder	1	4
Forearm	3	13
Elbow	1	4
*Initial clinical presentation* b		
Papule	9	39
Nodule	3	13
Cellulitis	3	13
Cellulitis & Ulcer	3	13
Ulcer	5	22
*Treatment*		
Surgical treatment alone	3	13
Medical treatment alone	4	17
Combination therapy	16	70
*Time to diagnosis (days) - median*		
Time from symptom onset to diagnosis	71	
Time from medical attention to diagnosis	35	

a Three cases had 2 single visit exposures to endemic areas. The causative single visit endemic area exposure was determined via VNTR sequencing.

b Clinical presentation was described by patient as the most prominent clinical presentation. Oedematous form was noted in two of the patients with cellulitis.

The lesions occurred primarily on the extremities, with the lower limbs the most prevalent site (14; 60%). Fifty-one percent of patients recalled mosquito bites within endemic regions, whilst only one patient reported having an open wound. The first medical professional sought for diagnosis and management was a general practitioner from a non-endemic area in 20 of the 23 cases (87%), with the diagnosis of BU suspected in only 17% of cases. The diagnosis of BU was suspected in 3 of 23 (13%) patients on first presentation overall, and 3 of 20 (15%) patients presenting initially to a non-endemic region general practitioner. The median time from symptom onset to PCR confirmed diagnosis was 71 days (range: 23–204 days). The median time from initial medical attention to diagnosis was 35 days (range: 15–103 days) for all patients.

Treatment was multimodal (medical management & surgery) in 16 (70%), surgery alone in 3 (13%) and medical alone in 4 (17%). A regime containing rifampicin and one of either clarithromycin or moxifloxacin was used in 17 (94%) patients whose medical management included antibiotic therapy. No episodes of relapse or new infection were reported after treatment completion. Follow-up ranged from 3 months to 9 years (median 24 months).

**Figure 3:**
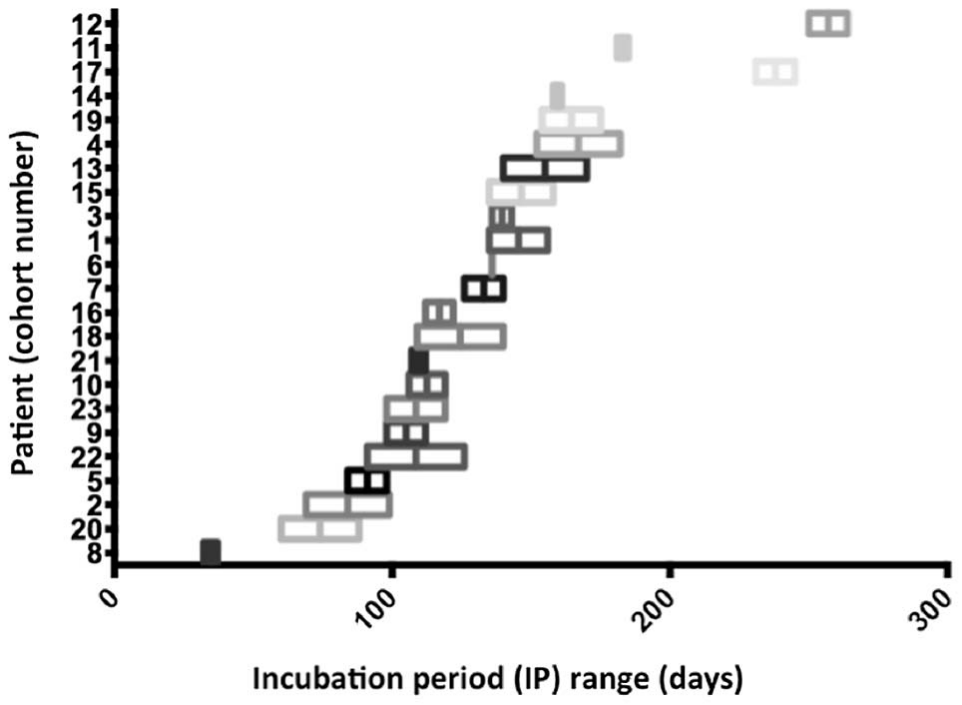
The IP for BU infection in the study population. The IP range (X-axis) for each patient (Y-axis) is demonstrated, arranged from shortest to longest IP. IP range midpoint values are marked as the middle line of each bar.

The midpoint of the reported IP range was utilized to calculate a point estimate of the IP for each case (Figure 3a). The IP point estimates for the 23 patients were normally distributed. The mean IP point estimate for the cohort was 135 days (IQR: 109–160 CI 95%: 113.9–156), corresponding to 4.5 months. The shortest incubation period noted was 32 days (1 month) and longest 264 days (9 months). Univariate analysis (Mann-Whitney U) did not identify any significant association between incubation period and sex (p = 0.489), endemic exposure (Bellarine Peninsula vs. other, p = 0.659), location of lesion (arm vs. leg, p = 0.391), age (≤30 vs. ≥31 years, p = 0.644) or the duration of exposure (<7 vs. >7 days, p = 0.207). (Table 3)

**Table 3:** The IP according to variables investigated.

Variable	Incubation period (IP)			
	Min (d/m)	Max (d/m)	Mean (d)^a^	Range (m)^b^
*Notification date*				
2003–2006	103/4	126/43	114	2–9
2007–2009	124/4	129/5	127	4–6
2010–2012	135/6	157/6	146	1–9
*Age (M = 30)*				
0–10	153/5	163/6	158	4–9
10–18	102/4	116/4	109	2–4
18–30	118/4	140/5	129	3–7
30–60	109/4	124/4	117	1–6
>60	140/5	170/6	155	4–9
*Sex*				
Male	135/5	143/5	139	1–9
Female	112/4	131/5	122	2–7
*Endemic*				
Bellarine	130/5	140/5	135	2–9
Mornington	135/5	158/6	147	1–9
Phillip Island	98/4	112/4	105	3–4
Gippsland	154/6	175/6	165	N/A
NT	140/5	170/6	155	N/A
NQLD	106/4	109/4	108	N/A
*Duration (d)*				
Single	124/4	129/5	127	1–9
1–7	122/4	131/5	127	4–7
7–14	158/6	172/6	165	3–9
>14	126/5	140/5	133	3–6
*Lesion locations*				
Arm	69/2	99/4	84	N/A
Leg	109/4	140/5	125	4–7

**Abbreviations:** Bellarine, Bellarine Peninsula; Mornington, Mornington Peninsula; NT, Northern Territory; NQLD, Northern Queensland; X, median; M, mean; d, days; m, months.

a **Mean**: The mean IP for each variable.

b **Range**: Shortest and longest incubation period recorded for specific variable,

## Discussion

Limited published data exists on the incubation period of Buruli ulcer. The Uganda Buruli Group in 1971 proposed an IP of 4–10 weeks in refugees who continued to develop BU following departure from an endemic refugee camp [17], with a rapid cessation of new diagnoses beyond 10 weeks. This may have been an underestimate, as the duration of exposure at the refugee camp is not entirely defined. A study of thirteen cases of BU from Port Moresby identified the shortest known incubation period (2–3 weeks) in a 6-week old baby born in an endemic region [18].

Historical data suggest incubation period estimates of 4–14 weeks [1] [13] [Hayman J, personal communication]. Reports in travelers to endemic regions have also aided research, such as a minimum IP of six weeks in a Nigerian physician working in New York City [25] and 5 months in a traveller from PNG [26]. Molecular typing has been used to identify region-specific *M. ulcerans* strains, from this the IP for two patients was estimated to be 3 and 7 months [17] and in five travelers from non-endemic areas to QLD and NT a range of 2 to 5 months was estimated [19].

This is the first public health investigation to systematically investigate IP in patients for whom exposure can be reasonably defined. Generally, it is not possible to do this as most patients with BU live permanently in endemic areas, hence time of transmission cannot be determined. Due to the previous systematic mapping documentation of endemic areas in Victoria and interviews with patients obtained through enhanced surveillance after notification we have estimated the mean IP for BU of 135 days, corresponding to 4.5 months or 19.2 weeks. The point estimates appeared normally distributed which suggests, but does not prove, that latency is not a major feature of *M. ulcerans* infection as we would expect a more right shifted distribution if this were the case, and that inoculum size is relatively similar between patients. The shortest IP noted was 1 month, the longest 9 months. We found no statistical association between individual IPs and other recorded covariables, but acknowledge this may be explained by our small final cohort size.

A secondary finding was of that BU is frequently not considered in the differential diagnosis when patients with BU first present to their doctor outside a BU endemic area. Buruli ulcer is an uncommon disease and diagnosis is often delayed when doctors are not familiar with the conditions. Diagnostic delay was not formally assessed with a matched cohort presenting in endemic areas in this investigation, however prolonged time to diagnosis in endemic compared with non-endemic areas has been reported previously [25].

This enhanced surveillance report has several limitations. The small final cohort number of patients in the group with single exposure to endemic areas limited the ability to determine meaningful associations with variables noted in Table 3. Furthermore, patient recall remains an issue particularly for earlier notifications. Nonetheless, the ability to cross-reference the telephone interview with the primary notification and enhanced surveillance forms reduced recall bias. In any investigation of BU, will always be an issue as there is a substantial period between exposure, symptom onset and diagnosis. Furthermore, as the mode of *M.ulcerans* transmission and exact inoculum size remains unknown, the impact of these variables is not completely understood. Nonetheless, the incubation period appears normally distributed around the point which suggests but does not prove that the inoculum size may have been similar in these 23 patients. We also acknowledge that the IP we have obtained was established in patients from Victoria only, and may or may not be generalizable to tropical Australia or the major endemic foci in sub-Saharan Africa. However our results are broadly similar to previous estimates from Africa and elsewhere.

### Conclusion

BU remains a significant cause of morbidity and disease in the developed and developing word. Defining the IP, 4.5 months (R: 1–9 months), provides a reference for future research in *M. ulcerans* epidemiology, pathogenesis and public health surveillance.
